# Stable isotopes in the shell organic matrix for (paleo)environmental reconstructions

**DOI:** 10.1038/s42004-023-01076-0

**Published:** 2024-01-18

**Authors:** Dragana Paleček, Stefania Milano, Igor Gutiérrez-Zugasti, Sahra Talamo

**Affiliations:** 1 Department of Chemistry “Giacomo Ciamician”, Alma Mater Studiorum-University of Bologna, Via Selmi 2, 40126 Bologna, Italy; 2 Leibniz Institute for Zoo and Wildlife Research (IZW), Department of Evolutionary Ecology, Alfred-Kowalke-Straße 17, 10315 Berlin, Germany; 3 Instituto Internacional de Investigaciones Prehistóricas de Cantabria (IIIPC), Universidad de Cantabria, Gobierno de Cantabria, Banco Santander, Av. de los Castros s/n, 39005 Santander, Cantabria Spain

**Keywords:** Biogeochemistry, Mass spectrometry, Environmental monitoring, Marine chemistry

## Abstract

Stable isotope ratios of mollusc shell carbonates have long been used to reconstruct past environmental conditions. Although shells also contain organics, they are seldom used in (paleo)climatic studies. Here, we extract the acid-soluble and insoluble fractions of the organic matrix of modern *Mytilus galloprovincialis* shells from three sites along a coast–to-upper-estuary environmental gradient to measure their hydrogen (δ^2^H) and oxygen (δ^18^O) isotope compositions. Both organic fractions showed isotopic signatures significantly different from those of carbonate and water at each site, indicating the involvement of different fractionation mechanisms. The soluble fraction showed gradual differences in isotope values along the gradient, while the insoluble fraction showed δ^2^H-δ^18^O correlation regressions subparallel to the Global and Local Meteoric Water Lines. These results showed the great potential of the shell organic matrix stable isotopes as possible (paleo)environmental proxies, stimulating further research to better define the fractionation mechanisms involved.

## Abstract (web summary)

Isotope ratios of mollusk shell carbonates are commonly used to reconstruct past environmental conditions, but the shell organic matrix is often overlooked. Here, the authors find the hydrogen and oxygen isotope compositions of the organic matrix of modern *Mytilus galloprovincialis* shells from sites along a coast-to-upper-estuary environmental gradient reflect environmental variables.

## Introduction

During their lifetime, molluscs incorporate environmental information in their shells in the form of chemical and physical properties. This allows for the use of seasonal and sub-seasonal shell growth increments to place the shell record into a temporal context and obtain information on local environmental conditions with a high temporal and geographical resolution (sclerochronology), similar to the analysis of annual growth rings in trees (dendrochronology)^1–6^. In archeological studies, sclerochronology can help study past environmental changes in connection with human activities and evolution^4^. However, beyond the importance of paleoclimatology and archeology, information recorded in mollusc shells is of increasing relevance for ecology and management^1,7^. It can be used to provide insights into the life history, population connectivity, productivity, and understanding the impacts of natural and anthropogenic environmental and climate change^1,7^. In sclerochronology, a number of proxies (i.e., stable isotopes and trace elements) are used to reconstruct different parameters of the (paleo)environment^1^. The use of stable isotopes as geochemical proxies relies on isotope fractionation during different chemical reactions (e.g., carbonate precipitation in molluscs) and the preservation of the resultant stable isotope ratio in the shell material^1–5^.

Among the different stable isotope ratios measured in mollusc shells, one of the most used and studied is the δ^18^O value, as an established water (paleo)thermometer^5,8–14^. The research involving different stable isotopes has so far been almost exclusively focused on the carbonate fraction of shells^1^. However, shells, being biogenic structures, are also composed of a small portion of organic matter (<5 wt%)^15–27^. The few available studies on stable isotopes focusing on the organic fraction use δ^13^C and δ^15^N as proxies for eutrophication and food web reconstruction^28–32^. So far, there is only one available study focusing on the potential use of the organic matrix stable isotope ratios for paleoenvironmental studies^33^. The advantage of using the shell organic matrix is the possibility to add the hydrogen isotope composition (δ^2^H) to the δ^18^O values, thus obtaining further information on shell deposition processes and how they may be affected by the environmental parameters. Furthermore, as opposed to the carbonates, the intracrystalline organic matrix is a closed system that was found to be isolated from the environment^34^ making isotopic exchange between the atmosphere and the intracrystalline organic matrix after deposition unlikely. Therefore, the stable isotope composition of the shell organic matrix could be a useful addition to the suite of available paleoenvironmental proxies.

The δ^2^H values of animals are often used to determine their trophic level and geographic origin since they reflect the animal’s diet^35,36^. Moreover, as mentioned, δ^18^O values in molluscs are an established proxy for water temperature, given the temperature-dependent isotope fractionation which occurs during the incorporation of oxygen into calcium carbonate^5,8–14^. Shell δ^18^O values have been demonstrated to be a function of water δ^18^O values, which in turn depends on local temperature and salinity variations^5,14^ and global (glacial) factors. In open ocean environments where there are no significant variations in salinity, temperature is the major controlling factor of shell δ^18^O values^5^. However, when considering estuarine species, it becomes necessary to take salinity into consideration as it also has a large effect on δ^18^O values^5^. The most successful applications in isotope studies involve multiple proxies, especially if the accompanying proxies provide information on main isotope controls, helping deconvolute multiple forcings such as these^5^. Due to the strong relationship between δ^2^H and δ^18^O values^37^, hydrogen isotopes could offer an additional proxy for the interpretation of (paleo)environments. A global empirical linear relationship for δ^2^H and δ^18^O values in precipitation was established called the Global Meteoric Water Line (GMWL), with a slope of 8 and an intercept of 10^37^. The relative fractionations of hydrogen and oxygen isotopes between water vapor and liquid water are partially reflected in the slope and intercept values. Based on the local conditions, these values can vary, with the fractionation changing in relative humidity values under 100% and at different temperatures, creating Local Meteoric Water Lines (LMWL)^38–41^. The δ^18^O value of precipitation in places with low slope values showed a positive correlation to monthly mean air temperature and a negative correlation to monthly mean precipitation^41^. Therefore, measuring both δ^2^H and δ^18^O values of the shells and examining their relationship could be useful for (paleo)environmental reconstructions, adding δ^2^H values as an additional proxy for surface water temperature reconstructions and as a potential provenance proxy. As the carbonate mineral phase does not contain hydrogen, the shell organic matrix can be used instead. Measurements of δ^2^H values in the organic matrix of freshwater species in relation to δ^18^O values of the shell showed promising results for future environmental reconstructions^33^. It was shown that the isotopic compositions of the organic matrix reflected the values of the water in which the shells lived. However, the authors did not discriminate between the acid-soluble and insoluble organic matrix fractions (SOM and IOM, respectively). These two organic matrix fractions were identified and differentiated in the early studies of the organic matrix composition of mollusc shells, both by their chemical composition and functions^16,19–22,26,42,43^.

**Fig. 1:**
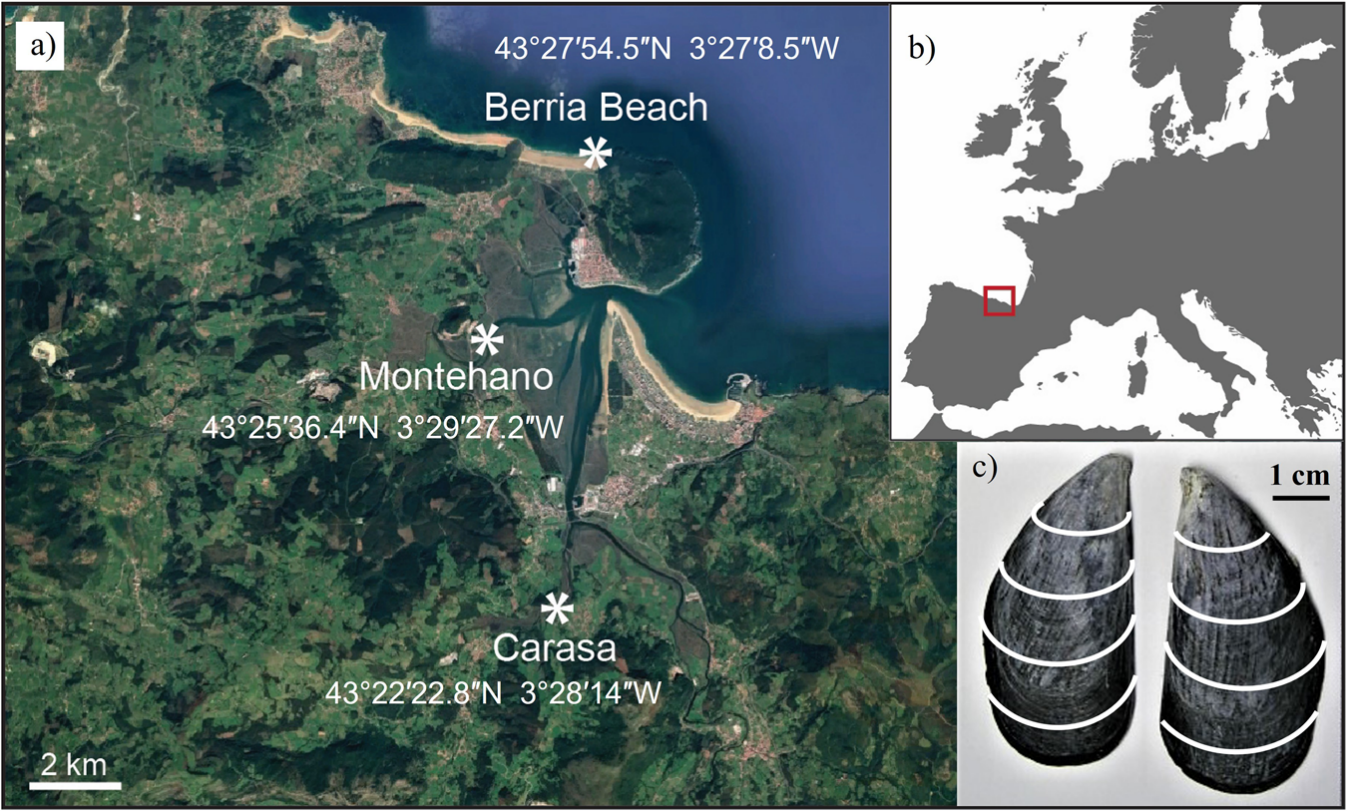
Map of the study localities. **a** Map of the three localities analyzed in this study: Berria Beach (marine habitat), Montehano (lower estuarine habitat) and Carasa (upper estuarine habitat). **b** Location of the studied area. **c** An image of a *M. galloprovincialis* sample from Carasa, with white approximate lines along which the samples were cut, dividing them into 2 to 7 subsamples based on the sample size. Figure modified after Milano et al.^48^.

In this paper, hydrogen and oxygen stable isotope measurements, performed on both the SOM and IOM of modern specimens of *Mytilus galloprovincialis* shells, were used to determine to what degree their values and relationship reflect the environmental variables of the water. *M. galloprovincialis* is a species with a wide geographical distribution^44–47^ and it was validated as a reliable species for the estimation of seasonal temperature fluctuations, as well as a potential provenance proxy using the correlation between carbon and oxygen stable isotopes^9,48^. The specimens for this study were collected from three sites along a coast-to-upper-estuary gradient in Northern Spain (Fig. 1) to evaluate if the SOM and IOM δ^2^H and δ^18^O values and their correlations vary along this gradient. The salinity is significantly different among the three sites, gradually decreasing from the coastal site (Berria) to the lower and upper estuary (Montehano and Carasa, respectively)^48^. Although there is no significant difference in surface temperatures among the sites, an increase in the temperature range from the coast to the upper estuary can be observed (ref.^48^; See Supplementary Notes 2 and 3, Supplementary Figs. S3 and S4). In this study, we compare the stable isotope measurements of the acid-soluble and insoluble organic matrix among the two fractions and between the three sites. Moreover, we compare the SOM and IOM δ^18^O values to previous δ^18^O measurements of the water and carbonates from *M. galloprovincialis* specimens collected at the same sites^48^. We hypothesize that the two organic matrix fractions have different isotopic signatures and thus reflect environmental variables in different amounts. Furthermore, we aim to determine to what extent measuring hydrogen isotopes along with oxygen isotopes contributes to a better understanding and interpretation of the local environmental conditions. The results of this study will help evaluate the potential of the stable isotope signatures of the SOM and IOM as proxies of the hydrographic characteristics of modern and ancient environments.

## Results

### Shell organic matrix stable isotope values

**Fig. 2:**
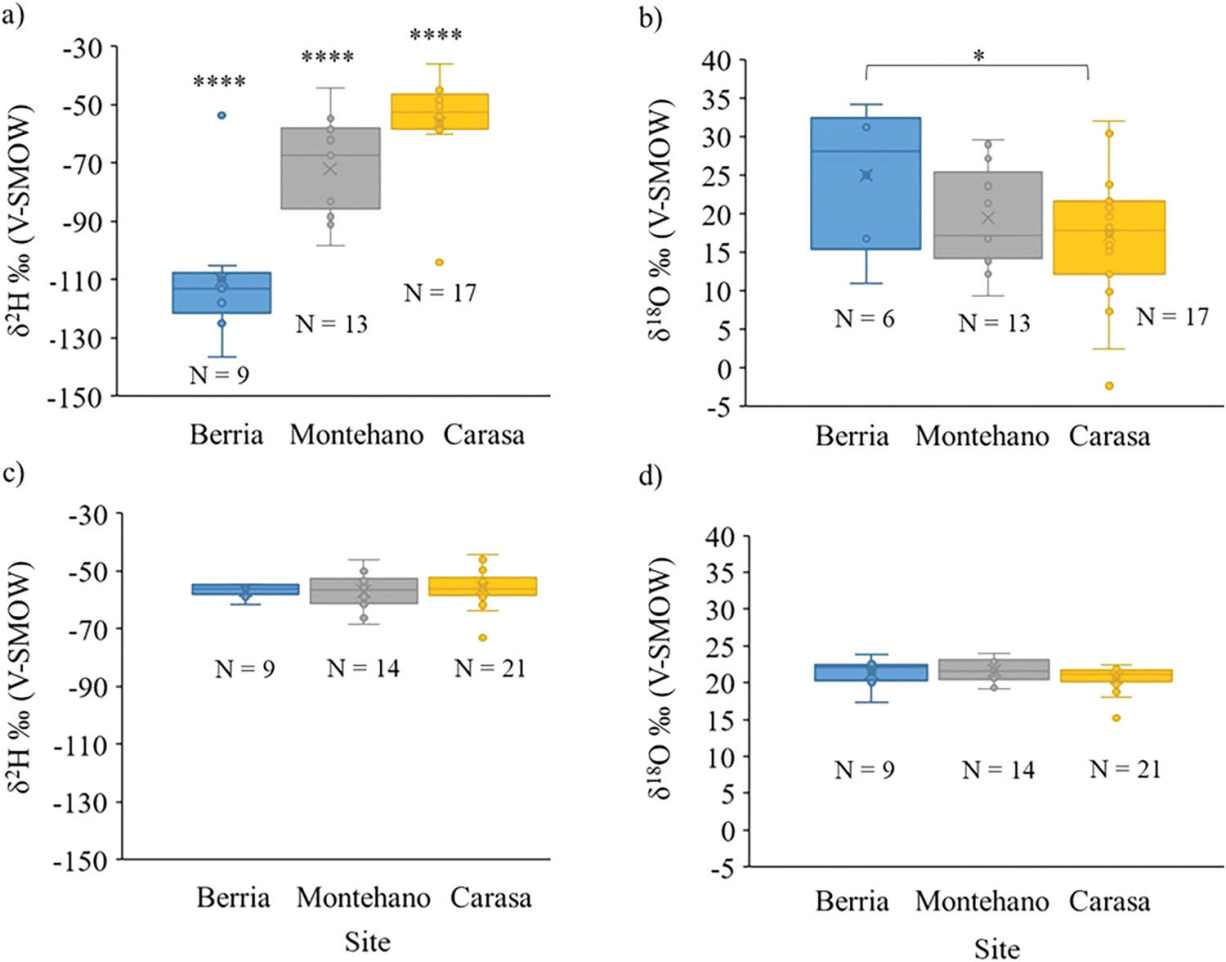
Hydrogen and Oxygen stable isotope values in the soluble and insoluble organic matrix fractions. Boxplots showing the ranges of **a** δ^2^H of the Soluble Organic Matrix (SOM); **b** the δ^18^O of the SOM; **c** the δ^2^H of the Insoluble Organic Matrix (IOM) and **d** the δ^18^O of the IOM. The number of samples in each site is indicated as *N*, **** = *p* < 0.0001; * = *p* < 0.05. The x represents the mean, the median value is shown as the line and outliers are given as the points outside of the whisker ranges.

**Fig. 3:**
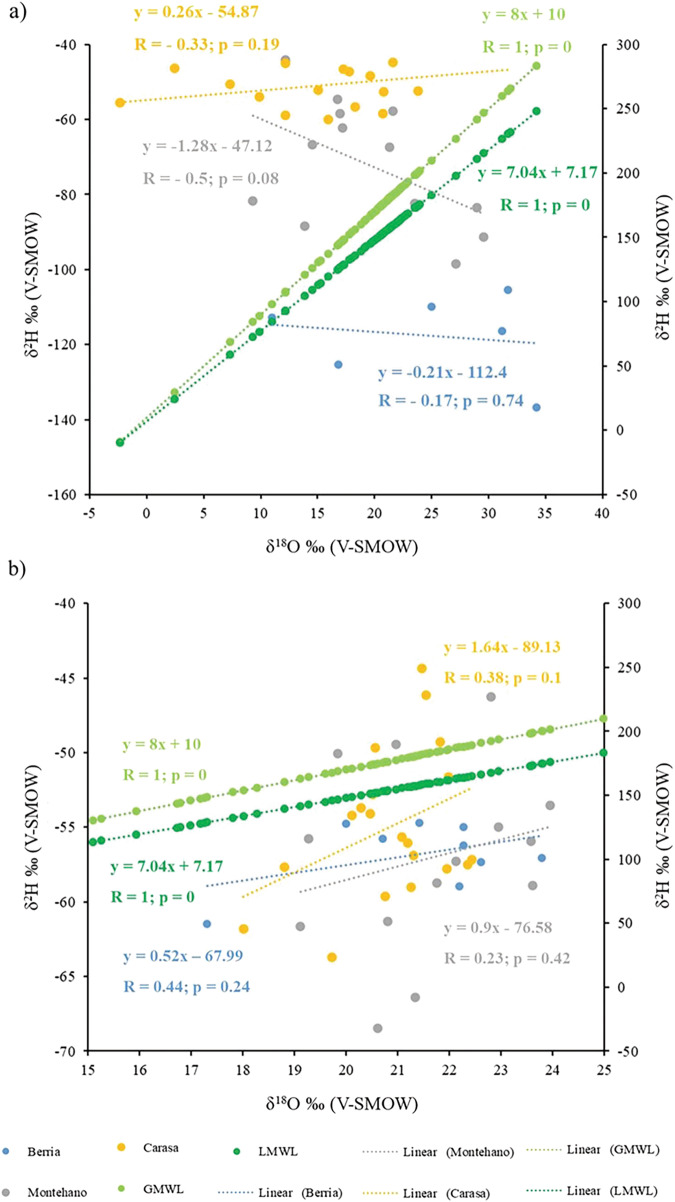
Hydrogen and oxygen isotope values of the soluble and insoluble organic matrix fraction compared to the global and local meteoric water lines. δ^18^O and δ^2^H (left *Y*-axis) of the **a** Soluble Organic Matrix (SOM) and **b** Insoluble Organic Matrix (IOM) compared to the GMWL and LMWL (δ^2^H of GMWL and LMWL right Y-axis).

The δ^2^H and δ^18^O values for the SOM and IOM from the three study sites are shown in Table 1. One outlier in the IOM and two outliers in the SOM from Carasa, as well as one outlier in the SOM from Berria (italic bold in Table 1), were identified and excluded from further data analyses. The ranges of δ^2^H in the SOM differed significantly between the three sites (p < 0.0001 Kruskal-Wallis, Fig. 2). The lowest δ^2^H values were observed in the SOM of Berria and the highest in Carasa, with Montehano showing intermediate values. The δ^18^O values of the samples from Berria were significantly different from those of Carasa (p < 0.05 ANOVA, Fig. 2), while samples from both Berria and Carasa showed similar ranges as samples from Montehano (p > 0.05 ANOVA, Fig. 2). For δ^18^O values in the SOM the highest values were observed in Berria, while the lowest were found in Carasa, showing an opposite trend compared to δ^2^H values. δ^18^O and δ^2^H values of the SOM showed a weak (Berria) to moderate (Montehano) negative linear relationship (Pearson’s R from −0.17 to −0.5, Fig. 3), while for Carasa, the δ^18^O and δ^2^H values showed a weak positive correlation (Pearson’s R 0.33, Fig. 3). However, the correlations were not statistically significant (p > 0.05). The δ^2^H and δ^18^O values of the IOM were similar among the studied localities (p > 0.05 ANOVA, Fig. 2). A weak to moderate linear relationship was observed between the δ^2^H and δ^18^O values in the IOM (Pearson’s R from 0.23 to 0.44, Fig. 3) which were not statistically significant (p > 0.05). The strongest relationship was observed in Carasa (upper estuary) and the weakest in Montehano (lower estuary). The δ^2^H values of the SOM differed significantly from the δ^2^H values of the IOM at all three sites (*t*-test Berria: p < 0.0001; Montehano: p < 0.01; Carasa: p < 0.05). The δ^18^O values of the SOM were significantly different from the δ^18^O values of the IOM at Carasa (*t*-test p < 0.01), while the differences between δ^18^O values of the SOM and IOM at Berria and Montehano had no statistical significance (*t*-test p > 0.05). Shells from Montehano and Carasa, which had enough material to be cut into several subsamples (see detailed explanation in the “Methods” section), showed a trend of lower values at the umbo toward slightly higher values at the ventral margin of the shells for δ^2^H values of both the SOM and IOM (Table 1, Supplementary Note 1, Supplementary Figs. S1 and S2). The trend was best observed in the largest sample from Carasa, which was divided into 7 subsamples (See “Methods” section and Supplementary Information for details). However, it was not as clearly developed in the δ^18^O values (Table 1, Supplementary Note 1, Supplementary Figs. S1 and S2).

**Table 1:** Stable isotope measurements. δ^18^O (‰) and δ^2^H (‰) values for the SOM and IOM.

	Soluble fraction (SOM)			Insoluble fraction (IOM)		
Site	ID	δ^2^H	δ^18^O	ID	δ^2^H	δ^18^O
Berria	6005.1.2	−112.7	11.0	6005.1.1	−56.2	22.3
Berria	6006.1.2	−118.0	n/a	6006.1.1	−61.5	17.3
Berria	6007.1.2	−113.2	n/a	6007.1.1	−54.8	20.0
Berria	6008.1.2	−125.2	16.8	6008.1.1	−57.3	22.6
***Berria***	***6003.1.2***	***−53.8***	***n/a***	6003.1.1	−55.8	20.7
Berria	6004.1.2	−136.7	34.2	6004.1.1	−55.0	22.3
Berria	6009.1.2	−109.9	25.0	6009.1.1	−54.7	21.4
Berria	6010.1.2	−105.3	31.7	6010.1.1	−59.0	22.2
Berria	6011.1.2	−116.5	31.2	6011.1.1	−57.0	23.8
Montehano	5993.1.2	−91.3	29.6	5993.1.1	−55.9	23.6
Montehano	5993.2.2	−54.7	16.7	5993.2.1	−53.5	24.0
Montehano	5993.3.2	−98.6	27.1	5993.3.1	−57.3	22.1
Montehano	5995.1.2	−82.4	23.6	5995.1.1	−58.8	21.7
Montehano	5995.2.2	−81.8	9.3	5995.2.1	−61.6	19.1
Montehano	5995.3.2	−88.5	13.9	5995.3.1	−50.1	19.8
Montehano	5995.4.2	−66.7	14.5	5995.4.1	−55.8	19.3
Montehano	5996.1.2	−62.2	17.2	5996.1.1	−66.4	21.3
Montehano	5996.2.2	−57.7	21.6	5996.2.1	−58.9	23.6
Montehano	5994.1.2	−83.4	29.0	5994.1.1	−61.3	20.8
Montehano	5994.2.2	n/a	n/a	5994.2.1	−49.4	21.0
Montehano	5994.3.2	−67.3	21.3	5994.3.1	−46.3	22.8
Montehano	5997.1.2	−58.5	17.0	5997.1.1	−68.4	20.6
Montehano	5997.2.2	−44.2	12.2	5997.2.1	−55.0	22.9
Carasa	6001.1.2	−56.6	18.3	6001.1.1	−57.8	21.9
Carasa	6001.2.2	−52.2	15.1	6001.2.1	−51.6	22.0
Carasa	6001.3.2	−48.4	19.6	6001.3.1	−53.7	20.3
Carasa	6001.4.2	−44.9	12.2	6001.4.1	−49.3	21.8
Carasa	6001.5.2	−47.1	17.8	6001.5.1	−52.8	20.5
Carasa	6001.6.2	−46.5	17.3	6001.6.1	−46.2	21.5
Carasa	6001.7.2	−35.9	32.0	6001.7.1	−44.4	21.5
***Carasa***	***5998.1.2***	***−104.2***	***30.6***	5998.1.1	−59.0	21.2
Carasa	5998.2.2	−52.5	20.8	5998.2.1	−54.2	20.1
Carasa	5998.3.2	−44.9	21.6	5998.3.1	−57.5	22.3
***Carasa***	***5999.1.2***	***−102.7***	***30.3***	***5999.1.1***	***−73.1***	***15.2***
Carasa	5999.2.2	−58.5	20.7	5999.2.1	−57.2	22.4
Carasa	5999.3.2	−52.4	23.8	5999.3.1	−56.9	21.3
Carasa	6000.1.2	−55.6	−2.3	6000.1.1	−59.6	20.7
Carasa	6000.2.2	n/a	n/a	6000.2.1	−54.1	20.5
Carasa	6000.3.2	n/a	n/a	6000.3.1	−57.7	18.8
Carasa	6000.4.2	−46.3	2.4	6000.4.1	−49.7	20.6
Carasa	6002.1.2	−58.8	12.2	6002.1.1	−63.7	19.7
Carasa	6002.2.2	−60.1	15.9	6002.2.1	−61.8	18.0
Carasa	6002.3.2	−53.9	9.9	6002.3.1	−55.6	21.1
Carasa	6002.4.2	−50.6	7.3	6002.4.1	−56.1	21.2

Missing values from samples with insufficient material for analysis are shown as n/a, and outliers in italic bold.

The results indicated that the two fractions have different isotopic signatures, suggesting the involvement of different fractionation mechanisms and therefore different influences from environmental and biological factors and possibly thermodynamic processes during biomineralization.

### Shell organic matrix, global meteoric water line and local meteoric water line

The relationship between meteoric water δ^18^O and δ^2^H values are shown in Fig. 3 and represented by the equations for the GMWL (Eq. (1)) and the LMWL of Santander (ca. 40 km from the study sites, Eq. (2))^37,39^:

1 $${{{{{\rm{\delta }}}}}}^{2}{{{{\rm{H}}}}}=8\times {{{{{\rm{\delta }}}}}}^{18}{{{{\rm{O}}}}}+10 \, {{{{\rm{for}}}}}\; {{{{\rm{the}}}}}\; {{{{\rm{GMWL}}}}}$$

2 $${{{{{\rm{\delta }}}}}}^{2}{{{{\rm{H}}}}}=7.04\times {{{{{\rm{\delta }}}}}}^{18}{{{{\rm{O}}}}}+7.17 \, {{{{\rm{for}}}}}\; {{{{\rm{the}}}}}\; {{{{\rm{LMWL}}}}}\; {{{{\rm{of}}}}}\; {{{{\rm{Santander}}}}}$$

The δ^18^O and δ^2^H values are inversely correlated in the SOM compared to the GMWL (Fig. 3). The slopes of the relationships in the SOM from the coastal site (Berria) toward the upper estuary (Carasa) do not show any clear trends. The regression lines of IOM δ^18^O and δ^2^H values are subparallel to the GMWL and LMWL (Fig. 3). Their slopes increase from the coast (Berria, slope = 0.5), to the lower estuary (Montehano, slope = 0.9) and toward the upper estuary (Carasa, slope = 1.6). The intercepts, on the other hand, decrease along the environmental gradient from the coast to the upper estuary (Berria, d = −68; Montehano, d = −77; Carasa, d = −89).

The results indicated that, even though the single isotope ratio values for the IOM did not show differences between the three study sites, the relationship between δ^18^O and δ^2^H values holds some environmental information. On the contrary, the relationship between the two isotope ratios in the SOM does not show any trend.

### Shell organic matrix isotopes and water isotopes

**Fig. 4:**
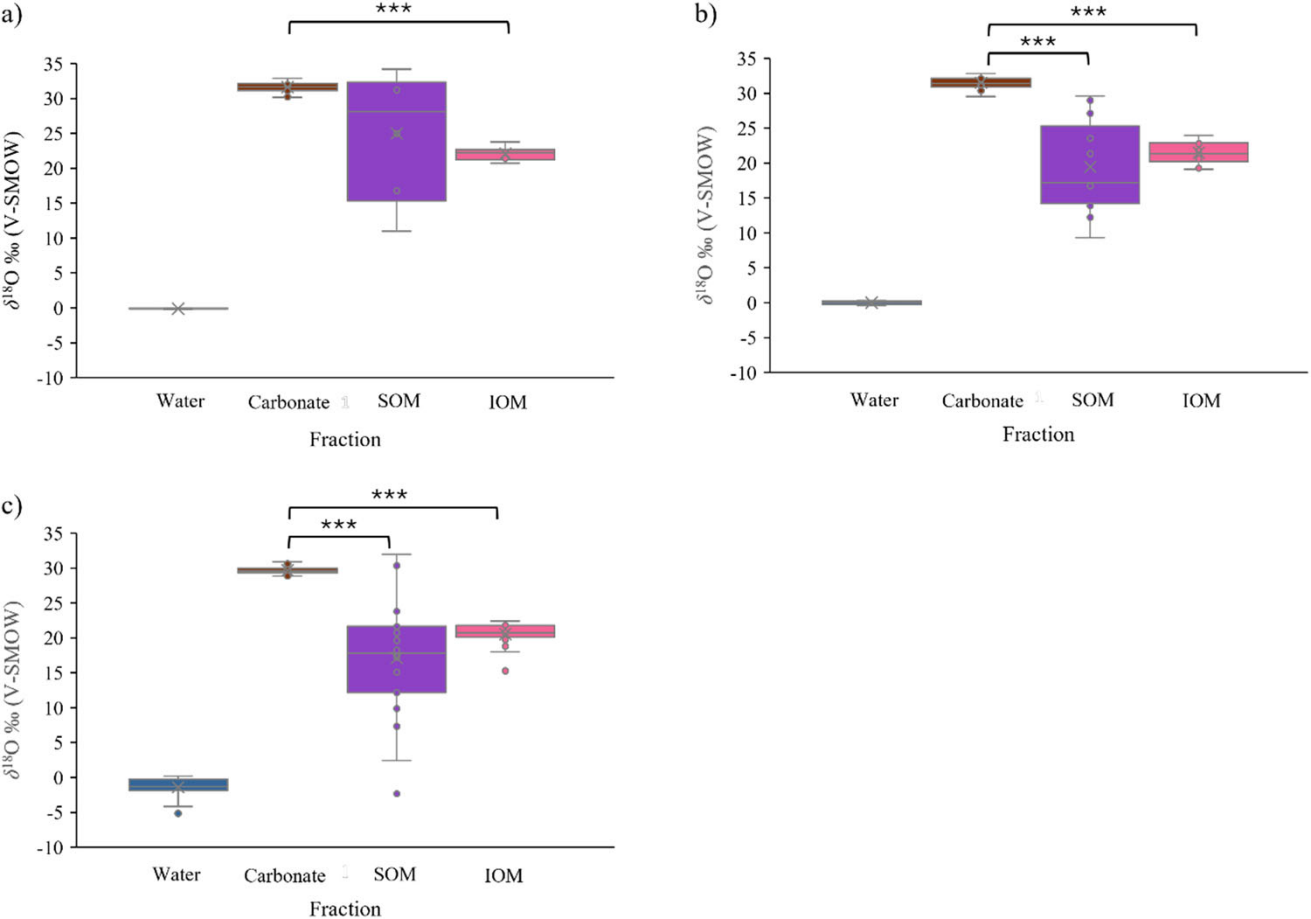
Comparison of the oxygen isotope values of the water, carbonate, and the soluble and insoluble organic matrix fractions. Boxplots showing the δ^18^O of the water, the shell carbonate as well as the Soluble Organic Matrix (SOM) and Insoluble Organic Matrix (IOM) phases for all samples from **a** Berria. N = 6; **b** Montehano. N = 13; **c** Carasa. N = 19. *** = p < 0.001. The x represents the mean, the median value is shown as the line and outliers are given as the points outside of the whisker ranges. Water and carbonate data from Milano et al. (ref. ^48^; Table 1; Page 69).

The δ^18^O values of the SOM and IOM differed significantly from respective water δ^18^O signatures at each site (Fig. 4). The SOM δ^18^O values showed a weak positive correlation with the δ^18^O values of water at Carasa (Pearson’s R = 0.2), while the correlation was negative at Berria (Pearson’s R = −0.1) and Montehano (Pearson’s R = −0.5). However, none of these correlations were statistically significant (p > 0.05). The IOM δ^18^O values showed a weak (Carasa, Pearson’s R = 0.1) to moderate (Berria and Montehano, Pearson’s R = 0.6 and 0.5, respectively) positive correlation with the δ^18^O isotope values of water from the same site. As for the SOM, none of the correlations were statistically significant (p > 0.05).

The results indicated that the organic matrix δ^18^O values differ significantly from both the carbonate and the water δ^18^O values available from a previous study^48^, indicating a substantial isotope fractionation in the organic matrix fraction.

## Discussion

In this study, the δ^2^H and δ^18^O composition of the acid-soluble and insoluble components of the mollusc shell organic matrix was analyzed. Here, we discuss the differences between the stable isotope ratios of the organic matrix and previous measurements on carbonates and water. Furthermore, we discuss the differences between the two organic matrix fractions and the three study sites observing the δ^2^H and δ^18^O values individually, after which we consider the differences in their relationship.

The δ^18^O measured in the organic matrix shows values that are significantly lower than the range of δ^18^O values in carbonates (Fig. 4). The only exception is represented by the mussels from Berria, in which the difference between the δ^18^O of the SOM and the carbonate is not statistically different. This possibly indicates a substantial isotope fractionation between the shell organic matrix (both IOM and SOM), the carbonates and the water where the molluscs were growing (Fig. 4). The differences in fractionation among the carbonates and the organic matrix are likely due to the formation mechanisms of these two phases. There are different hypotheses on the biomineralization processes in mollusc shells^15,49–51^. However, it is generally recognized that the mechanisms behind the deposition of the calcium carbonate and the organic matrix are different and take place successively. The formation of the organic framework precedes crystallization and is supplied by the calcifying secretory epithelium of the mantle^15^. The shell organic constituents were defined as a ‘matrix’ consisting of a mixture of extracellular macromolecular components secreted to guide the mineralization, acting as a substrate for mineral deposition^15^. In multicellular organisms, marine biominerals are formed within a biologically controlled compartment termed “privileged space”^52^. This space is bounded by a single layer of specialized epithelial cells containing extracellular calcifying fluid, which in molluscs takes the form of extrapallial fluid found between the mantle and the growing shell^52^. Depending on the species, it can be more or less open to the environment^52^, which could affect the degree of isotope fractionation. Although there are variations depending on taxon^53^, the general principles that govern the physiology of shell formation are valid for all calcifying molluscs^15,52^ and they involve a combination of physical and biological controls^52,53^. A review of the calcification processes in different taxa classified *Mytilus sp*. into a category of molluscs with microstructure organization in which crystal orientation is determined by interaction with organic matrices and in which the biomineralization process is highly influenced by physical, but also in a fair amount, by biological processes^53^. Therefore, during biomineralization in molluscs, the different mechanisms involved in the formation of the calcium carbonate and the organic matrix are possibly influenced by both biological and physical processes. Given the difference in isotopic signatures between the carbonates and the organic matrix, these influences also vary between these two phases.

Furthermore, the results show that the δ^2^H and δ^18^O isotope ranges differ significantly within the organic matrix. For instance, the SOM shows greater variability than IOM. In the SOM, we observe a gradient in δ^2^H starting from very negative values at the coast toward more positive values in the upper estuary (Fig. 2), which is opposite to the variation in δ^18^O and opposite to expected water composition depending on Rayleigh fractionation. This corroborates that the incorporation of hydrogen and oxygen in the two fractions may be influenced by different fractionation processes. They might be related to different dietary inputs, metabolic or enzymatic activity during biomineralization, or the different formation pathways and compositions of the SOM and IOM. Even though it was discovered that the two fractions share several proteins, which are the main building blocks of the shell organic matrix^15,22,24,54^, the SOM and IOM are biochemically and functionally distinct^17–21,27^. The main amino acids composing the proteins that make up the hydrophobic insoluble fraction were listed as glycine, alanine, phenylalanine, and tyrosine^15,18^. Furthermore, the cross-linking of proteins by the enzyme phenoloxidase makes this fraction acid insoluble^18^. On the other hand, the proteins of the soluble matrix were found to be rich in acidic hydrophilic residues, in particular aspartic acid and, to a lesser extent, glutamic acid^15,17,18^. However, the amino acid composition of the organic matrix proteins has been shown to vary among species and even specimens (throughout their life and in different shell layers)^24,25,54,55^. The functions performed by different matrix constituents are still poorly understood; however, it is clear that they have important roles in biomineralization^27^. The acid-insoluble matrix is thought to act as a structural framework, directing the calcium carbonate mineralization by regulating crystal nucleation, orientation, and polymorphism^18,20,21,27^. On the other hand, the acid-soluble fraction was found to have a role in the binding of calcium ions regulating crystal growth and morphology^18,20,21,27^. Even though the chemical composition of the two organic matrix fractions changes based on the shell microstructure, their functions remain constant^27^. Furthermore, the soluble matrix proteins were found to be mainly within biominerals, i.e., intracrystalline, while the insoluble matrix proteins were localized mostly around the crystal phase (‘intercrystalline’)^15^. However, in this study, the intercrystalline organic matrix was mostly removed during extraction, underlining the presence of both the SOM and IOM in the intracrystalline fraction analyzed here. These differences between the SOM and IOM might be the key to understanding the different stable isotope signatures observed in these two organic matrix fractions.

The differences between the three sites are demonstrated by the significant variation of δ^2^H values within the SOM. Since this variation follows the gradient from the coast to the upper estuary, it might be related to the evaporative processes and the salinity gradient. For instance, variations of shell δ^13^C in *Mytilus edulis* have been observed in relation to different salinity conditions^56^. Previous studies found that the composition of the extrapallial fluid of molluscs from marine and freshwater species forming aragonitic shells differed greatly, partially reflecting the solutes in the environment^57^. This might be related to the fact that osmoregulation in estuarine mollusc species causes changes in metabolic activities, including intracellular enzyme activity and amino acid regulation^58^. According to our data, the δ^2^H in IOM does not vary between the three sites while the SOM does, this might indicate that the SOM is more affected by these processes compared to the IOM. As previously stated, the organic matrix of mollusc shells is known to have a key role in biomineralization processes^17,20,59^ but is also thought to take part in cell signaling, enzymatic^15,24,25^ and immunity functions^60^, which might also affect isotope fractionation. During biomineralization, the ions required to construct the calcium carbonate are transported from the environment to the site of shell deposition or the extracellular calcifying fluid^52^. To complete the construction of the crystalline structure, a series of organic matrix–mediated processes is activated^57,61^. There are many possible “vital” or “physiological effects” for different proxies that are involved in the biomineralization process^57^. An example of such an effect is the activity of the carbonic anhydrase enzyme^15,57^. These enzymes catalyze the reversible hydration of metabolic carbon dioxide (CO_2_) to bicarbonate (HCO_3_^−^) requiring protons (H+)^62^, thus regulating the formation of calcium carbonate crystals in the shell. Changes in salinity were found to modify the activity of these enzymes, thus affecting the regulation of shell biomineralization^63^. The exact location where these processes occur is still unknown, although the activity of these enzymes was observed in the mantle, but also in the shell organic matrix^15^. Therefore, isotope fractionation in the organic matrix might be affected by this type of enzymatic activity, which in turn depends on the external conditions. Furthermore, given the differences in the SOM and IOM isotopic signatures, the enzymatic activity involved in biomineralization might mainly be concentrated in one of the two fractions causing the differences in isotope fractionation.

Alternatively, differences among the three sites in the diet and metabolism of the molluscs could be influencing isotope fractionation in the SOM. Hydrogen isotope ratios of marine snail soft tissues have been shown to be more influenced by their food source rather than ambient water^64^. However, this work was criticized, suggesting that isotope fractionation in animal tissues is most likely influenced by multiple factors, including diet and water^65^. Metabolic responses to stress caused by external environmental factors (temperature, salinity, nutrient availability) or by internal factors (reproductive stress or disease) can also cause changes in isotope fractionation in animal tissues^66^. For example, changes in salinity and temperature have been found to affect the metabolism and stress response of *Mytilus* sp.^67^. Furthermore, the biochemical composition of *M. edulis* tissues was found to vary due to different temperatures, phytoplankton availability and reproductive cycle^68^. Many bivalve species, including *Mytilus* sp., are filter feeders, acquiring their proteins, lipids, carbohydrates, and other components which are integrated into their tissues mainly from their diet^68–70^. Deuterium depletion in cellular organic matter during the metabolism of carbohydrates into lipids was observed in microalgae^71^. Thus, the consumption of microalgae with variable lipid content and/or the in-situ synthesis of lipids might affect the hydrogen isotope fractionation in *Mytilus sp*. Lipids constitute a lesser-known minor portion of the shell organic matrix and are thought to have a significant role in biomineralization^15,27,72–74^. The lipid composition of the organic matrix was found to vary among species and different structures within the shell (i.e., nacreous and prismatic; refs. ^73,74^), and their quantity is likely variable and possibly dependent on diet. Due to restrictions in terms of sample size, we were unable to analyze the aragonite and calcite layers separately in this study. Analyzing the different structures and polymorphs separately will allow us to obtain more information, assessing the potential influence of mineralogy and microstructure on the stable isotope signature of the organic matrix. To be able to obtain sufficient material for organic matrix extraction, it would be ideal to perform these analyses on a species containing only one carbonate polymorph (i.e., *Pecten* sp. such as *Pecten maximus* or *Crassostrea* sp. such as *Crassostrea gigas*). In fact, the organic matrix has been indicated for studies of the mollusc diet in early research and it showed similar δ^2^H values in *M. edulis* flesh and bulk organic matrix^16^. Consequently, isotope fractionation in the organic matrix of mollusc shells might also be influenced by multiple factors, including diet and metabolism. These factors are most likely variable along the coast-to-upper-estuary gradient due to different environmental conditions and phytoplankton availability, characteristic of estuarine habitats. For example, a study conducted on the River Pas estuary (ca. 40 km west to localities studied here and with similar environmental conditions) showed differences in phytoplankton abundance and diversity between the river mouth and upper estuarine area when considering the same time period^75^. They observed a productivity and diversity increase along the longitudinal axis of the estuary toward the sea^75^. Furthermore, the average size of the shells in the three sites increased from the coast to the upper estuary. This increase is most likely related to the different environmental conditions and phytoplankton availability in the three sites and has an impact on the molluscs’ diet and metabolism. Therefore, both the physical and chemical environmental variables and different phytoplankton communities available at the three study sites might have an influence on the isotopic signatures of the organic matrix of the shells.

Within the SOM, we observe a higher variability in δ^2^H values compared to δ^18^O (Fig. 2). This could be partially explained by the natural variation in the isotope ratios of these two elements, which is larger for δ^2^H due to the greater mass difference among hydrogen isotopes^39,40,66^. This variation makes it a high-resolution environmental proxy and it is one of the reasons for the frequent use of hydrogen isotope ratios in studies of animal origin and migration^76–81^ and, more recently, food web and trophic level studies^35,76,82,83^. In these studies, diet and ambient water are considered the main sources of hydrogen in animal tissues; however, the relative contributions of these sources are variable and still not clearly defined. Furthermore, the proportion of dietary versus synthesized lipids and amino acids in animal tissues is uncertain and would have an effect on the tissue δ^2^H^35^. This is likely true for mollusc shells as well. Overall, the observed values and variability of δ^2^H in the organic matrix highlight its potential as an environmental proxy, but further research will be needed to assess the impact of metabolic pathways on the δ^2^H in the SOM and how they differ from the IOM.

Apart from considering the stable isotope variation of oxygen and hydrogen individually, it is important to observe how their correlation changes as well. A previous study on *M. galloprovincialis* shells from the same three sites considered here found that the relationship between δ^18^O and δ^13^C of the carbonate phase could be used as a provenance proxy^48^. The relationship between δ^18^O and δ^13^C showed negative correlations in the marine environment and positive correlations in the estuarine environment^48^. A different study found that the δ^2^H of the organic matrix and the δ^18^O in the mineral fraction of the shell had a positive correlation with a slope subparallel to the GMWL^33^. This result suggests that the functional relationship between the oxygen and hydrogen isotopes found in meteoric water is maintained in the shell^33^.

Our results for the SOM fraction had a higher variability giving negative relationships between the δ^2^H and δ^18^O for Berria and Montehano and a positive relationship for Carasa, yet showing no trends correlated to the environmental gradient (Fig. 3). The different relationships are given by the opposite trends of δ^2^H and δ^18^O along the geographical gradient. Heavy isotope-enriched river water can be an effect of increased evaporation^84^ and/or the abundance of phytoplankton^85,86^. However, the variation of water δ^2^H and δ^18^O typically follows the same trend for both isotopes, from lower values at the river source to higher values at the river mouth^16,84,87^ or vice versa depending on rainfall and evaporation^88^. At the three study sites, the δ^18^O values of water are lower at Carasa and higher at Montehano and Berria^48^. Thus, in the case of SOM, δ^2^H values follow a trend that is opposite to what could be expected, supporting the hypothesis that hydrogen incorporation may be dependent on metabolism or diet and therefore possibly indirectly related to environmental variables.

On the other hand, the results for the IOM were comparable to those presented in the study by Carroll et al.^33^, with positive correlations and slopes subparallel to the GMWL and LMWL even though the correlation was not statistically significant (Fig. 3). This lack of statistical significance might be due to the limited sample size. However, the slopes and intercepts of the δ^2^H and δ^18^O relationships in the IOM change following the environmental gradient, indicating that they have a potential as an environmental proxy. The hydrogen and oxygen isotope compositions of meteoric water can form slopes as low as 4 and as high as 9 depending on the geographical position and thus local conditions forming LMWLs^38,41^. The LMWL for the area of study has a lower slope value of 7.04 and an intercept of 7.14 due to local conditions^39^. Therefore, as expected, it has an even closer value to the slope obtained for Berria compared to the GMWL (Fig. 3). Surface water δ^2^H and δ^18^O were found to depend mostly on the spatiotemporal variation in precipitation isotope ratios and runoff generation over large geographic areas^85,89^. However, there were some local deviations from precipitation isotope ratios due to elevation in mountain-fed rivers and due to evaporative effects in small streams^89^. For example, surface water in rivers in the British Isles showed a lower slope compared to the GMWL due to evaporative processes causing heavy isotope enrichment^90^. Thus, in future studies, it would be necessary to perform water δ^2^H measurements in the study sites in addition to δ^18^O. For this study, we had access to shell samples and preexisting water data, and unfortunately, the water δ^2^H data was not available. Nonetheless, the differences in slope and intercept values in IOM δ^2^H and δ^18^O relationships among the three sites indicate that, although the ranges of the single isotope values are limited, their correlation may hold some environmental information. Alternatively, the differences may be due to different food sources in the three sites, which in turn influence the organic matrix δ^2^H. Hence, we could assume that the differences between the IOM slopes among the three sites depend on the local conditions. It is known that the relationship between water δ^2^H and δ^18^O can change seasonally^90^. However, seasonal variation in river surface water was shown to be lower than in precipitation, indicating that the isotopic composition of river water was also influenced by the input of groundwater^85^. Regardless, it would be challenging to obtain seasonal data using the organic matrix from small shells such as *Mytilus* sp. Given the high amount of shell material needed for extraction (~2 g), using the organic matrix results in a lower number of subsamples which can be analyzed compared to isotope analysis of the carbonate phase^5,9,10,48^. However, studies on seasonality could target species with larger and thicker shells to obtain more subsamples, for example, *Pecten* sp. or *Crassostrea* sp., which would also allow for the study of potential ontogenetic effects at a higher resolution. Furthermore, extracting the organic matrix from different calcium carbonate structures and polymorphs separately would allow us to assess if there are any potential differences in stable isotope composition in the organic matrix.

## Conclusions

This study is the first attempt to investigate the variation of stable isotopes along a geographical gradient using the organic matrix of mollusc shells. The advantage of using the organic matrix fractions as opposed to the carbonate fraction is that it allows for the measurement of not only δ^18^O but also δ^2^H. Furthermore, this is, to the best of our knowledge, the first study to differentiate the measurements in the SOM and IOM. Our results demonstrate that the SOM shows a much higher variability in terms of δ^2^H and δ^18^O compared to the IOM. Given the differences among the two fractions of the organic matrix, future studies should maintain the division into the SOM and IOM and analyze them separately. Moreover, the δ^2^H ratios from the SOM could potentially be a new high-resolution provenance proxy, which might help to disclose information on the hydrographic characteristics of modern and ancient environments. However, further research is needed to better define the biochemical processes that affect the isotopic composition of the organic matrix. Furthermore, when evaluating δ^2^H and δ^18^O together, we observe positive correlations, although not statistically significant, in the IOM subparallel to those of GMWL and LMWL and whose slopes follow the coast-estuary gradient, indicating that environmental information might also be encoded in the IOM fraction. We suggest the inclusion of δ^2^H and δ^18^O measurements of water and phytoplankton isotope ratios in further studies. A separate analysis of the organic matrix from calcite and aragonite fractions, as well as different microstructures, would assess the existence of potential biases due to different calcium carbonate polymorphs and microstructures. Furthermore, the use of larger species with a longer life cycle would give the possibility of obtaining subsamples at a higher resolution which might reveal any possible ontogenetic trends. Our results show an overall potential in using shell organic matrix stable isotopes as proxies of local hydrological features. However, in order to apply these methods to archeological shells, it is important to consider the effects of diagenesis on the stable isotope composition of the organic matrix. In this study, the intracrystalline organic matrix fraction was used since it is expected to be protected from the influence of these processes. However, more research will be needed to clarify and further develop the application of our observations.

## Methods

### Shell collection and preparation

A total of 19 *M. galloprovincialis* shells were collected from three sites in Cantabria, Northern Spain (Fig. 1). Out of these, nine samples were collected from Berria Beach (43°27′54.50″N, 03°27′08.50″W), five from a lower estuarine site near Montehano (43°25′36.40″N, 03°29′27.20″W) and five from an upper estuarine site near Carasa (43°22′22.80″N, 03°28′14″W; WSG84). The sampling was performed aiming to collect the largest specimens available in order to have the most complete environmental record possible. Average shell sizes varied between the three study sites. Shells collected in Berria were the smallest, with an average size of 2.7 ± 0.3 cm ranging from 2.1 to 3.1 cm in length and an average weight of 1231.4 ± 451.6 mg ranging from 675 to 2075 mg. Shells from Montehano had an average length of 4.9 ± 0.8 cm with samples measuring from 4.2 to 5.8 cm, the average weight was 5929.6 ± 1360.2 mg with samples ranging from 4654 to 7781 mg. Finally, in Carasa, the shells were the largest on average, with 5.3 ± 0.4 cm in length and 8950.6 ± 3994.6 mg in weight, ranging from 5 to 5.9 cm and weighing from 5804 to 15496 mg. Soft tissues were removed immediately after collection, and the shells were air-dried and stored in polyethylene bags for further analyses. The insoluble and soluble fractions of the organic matrix were extracted in the Bologna Radiocarbon Laboratory devoted to Human Evolution (BRAVHO lab) employing the method routinely used on modern shells and coral samples in studies focusing on the organic matrix, requiring around 2–2.5 g of shell material for the extraction^91,92^. The shell samples were first physically cleaned with a mechanical drill to remove surface contaminants, and further impurities were chemically removed by immersing them in 5 vol% sodium hypochlorite solution overnight. After these cleaning steps, the shells were rinsed in MilliQ water several times to wash off the sodium hypochlorite and any loose debris and left to air-dry. Samples from Montehano and Carasa were large enough to cut into several subsamples close to 2 g, while the samples from Berria were left whole due to their smaller size (Fig. 1). The cutting was performed along the growth lines of the shells aiming to obtain information on changes in the isotope composition during shell growth (Fig. 1, SI Supplementary Note 1, Supplementary Figs. S1 and S2). All samples, containing both the aragonite and calcite layers, were hand-crushed to powder in an agate mortar and further crushed in an automatic mill to obtain a finer powder. The obtained powder was sieved with a 150 µm mesh stainless steel sieve, and the finer fraction was put into labeled glass tubes. The powdered samples were once again left in 5% sodium hypochlorite solution overnight for thorough removal of non-shell organic material, rinsed three times with MilliQ water and dried in the oven for 2 days at 60 °C. The second hypochlorite step removes most of the intercrystalline organic matrix of the shell, leaving the intracrystalline fraction, which should be unaffected by diagenesis and contamination when using archeological samples. The powder was then transferred into regenerated cellulose membranes for dialysis (MWCO = 3.5 kDa) with 5 ml of MilliQ water. The sealed membranes were then put into 1 L of 0.1 M CH_3_COOH solution under stirring. The solution was changed every 5 days until the samples were decalcified; subsequently, it was replaced by MilliQ water to reach a pH value of around 6. The obtained dispersion containing organic matter was centrifuged at 3500 rpm (revolutions per minute; 2301 × *g* units of gravity or times gravity) for 5 min to separate the soluble (liquid) and insoluble (solid) fractions. Both fractions were then lyophilized and weighed before further analysis.

### Stable isotope analysis

Measurements of stable isotope ratios were performed at the Leibniz Institute for Zoo and Wildlife Research (Leibniz IZW) in Berlin. Aliquots of 0.35 ± 0.10 mg from each sample were placed into silver capsules (IVA Analysentechnik e.K. Meerbusch, Germany) and then analyzed using a Delta V-Advantage mass spectrometer (Thermo Fisher Scientific, Bremen, Germany) connected via an interface (Conflo IV, Thermo Fisher Scientific, Bremen, Germany) to a High Temperature Conversion Elemental Analyser (TC/EA Thermo Finnigan) and an online temperature-controlled vacuum-equilibration autosampler Uni-Prep (EuroVector). Samples and reference materials were loaded into the autosampler at 60 °C. Measurements were performed using the comparative equilibration method to calculate the isotope ratio of the non-exchangeable portion of hydrogen^93^. After flushing with helium and evacuating the carousel, 20 μl of water of known isotopic composition was injected through the Uni-Prep septum for equilibration (1 h). The samples were measured together with three in-house keratin standards: sheep wool from Sweden SWE- SHE [δ^2^H = −111.65 ‰, δ^18^O = 10.84 ‰], sheep wool from Spain ESP- SHE [δ^2^H = −61.54 ‰, δ^18^O = 16.94 ‰] and goat wool from Tanzania AFR- GOA [δ^2^H = −26.44 ‰, δ^18^O = 22.29 ‰]. Stable isotope ratios (δ^2^H and δ^18^O) were expressed as deviations from the international reference material Vienna Standard Mean Ocean Water (V-SMOW). Measurement precision was always better than 1 ‰ for δ^2^H and δ^18^O (1 SD).

### Data analysis

All statistical data analysis was performed using the PAST 4.12 (PAleontological STatistics) software package^94^. For all data, outlier and normality tests were performed prior to further data analysis. To detect differences among the different localities, ANOVA and Kruskal-Wallis tests were used, along with appropriate post-hoc tests when necessary (Tukey’s and Mann-Whitney’s). Pearson’s correlation indices and significances for each of them were determined to demonstrate the presence or absence of correlation between variables. An alternative statistical analysis (GLMM) was also included in the Supplementary Information (Supplementary Note 4, Supplementary Fig. S5). Water and carbonate data from Milano et al.^48^ (Table 1; Page 69) were included in the data analysis presented here.

## Supplementary information

Supplementary Information

## Data Availability

Data are available through Mendeley Data at 10.17632/y9rpmd428x.1.
