# Author Correction: Stable isotopes in the shell organic matrix for (paleo)environmental reconstructions

**DOI:** 10.1038/s42004-025-01745-2

**Published:** 2025-10-28

**Authors:** Dragana Paleček, Stefania Milano, Igor Gutiérrez-Zugasti, Sahra Talamo

**Affiliations:** 1 Department of Chemistry “Giacomo Ciamician”, Alma Mater Studiorum-University of Bologna, Bologna, Italy; 2 Leibniz Institute for Zoo and Wildlife Research (IZW), Department of Evolutionary Ecology, Berlin, Germany; 3 Instituto Internacional de Investigaciones Prehistóricas de Cantabria (IIIPC), Universidad de Cantabria, Gobierno de Cantabria, Banco Santander, Santander, Cantabria Spain

Correction to: *Communications Chemistry* 10.1038/s42004-023-01076-0, published online 18 January 2024

In the version of this article initially published, there were errors in reporting earlier data (from Milano, S. et al. *The Holocene* 10.1177/0959683619865595 (2020)), affecting the carbonate plots, significance bars, and associated text in Figure 4. Figure 4 has now been updated.

In the first sentence of the “Shell organic and water isotopes” section of the Results, in the sentence now reading “The δ^18^O values of the SOM and IOM differed significantly from respective water δ^18^O signatures at each site (Fig. 4),” the sentence originally began with “Unlike the carbonates^48^…”.

In the second paragraph of the Discussion, the sentence now reading “The δ^18^O measured in the organic matrix shows values that are significantly lower than the range of δ^18^O values in carbonates (Fig. 4). The only exception is represented by the mussels from Berria, in which the difference between the δ^18^O of the SOM and the carbonate is not statistically different” replaces the original “The δ^18^O measured in the organic matrix shows values much higher than the range of δ^18^O values in carbonates, which are much closer to the water δ^18^O values (Fig. 4).”

These changes have been made in the HTML and PDF versions of the article. For comparison, the original Figure 4 is available as Supplementary Information alongside this amendment.

## Supplementary information

Original, uncorrected Fig. 4

