# Supplementary material for: Stable isotopes in the shell organic matrix for (paleo)environmental reconstructions
Source: Commun Chem. 2024 Jan 18;7:16. doi: 10.1038/s42004-023-01076-0 (PMC10796374; doi:10.1038/s42004-023-01076-0)
Supplement: Supplementary file 1 — Supplementary Information [file 42004_2023_1076_MOESM1_ESM.pdf]

Supplementary Information

Stable isotopes in the shell organic matrix for (paleo)environmental reconstructions

Dragana Paleček[1*], Stefania Milano[2], Igor Gutiérrez-Zugasti[3], Sahra Talamo[1]

[1] Department of Chemistry Giacomo Ciamician, Alma Mater Studiorum - University of Bologna, Via Selmi 2, 40126 Bologna, Italy

[2] Leibniz Institute for Zoo and Wildlife Research (IZW), Department of Evolutionary Ecology, Alfred-Kowalke-Straße 17, 10315 Berlin, Germany

[3] Instituto Internacional de Investigaciones Prehistóricas de Cantabria (IIIPC), Universidad de Cantabria, Gobierno de Cantabria, Banco Santander, Av. de los Castros s/n, 39005 Santander, Cantabria, Spain

Supplementary notes 1: Hydrogen and oxygen isotope ratios of subsamples from Montehano and Carasa

Supplementary notes 2: Time series of water physical data and geochemistry

Supplementary notes 3: Comparison of salinity, temperature, and oxygen isotope ratios among the three sites

Supplementary notes 4: Generalised Linear Mixed Model analysis - GLMM

Supplementary notes 1: Hydrogen and oxygen isotope ratios of subsamples from Montehano and Carasa

The following graphs show the stable isotope ratios of the subsamples from Montehano and Carasa, shown ordered from the first subsamples taken at the umbo region of the shells to the last subsample collected at the ventral margin.

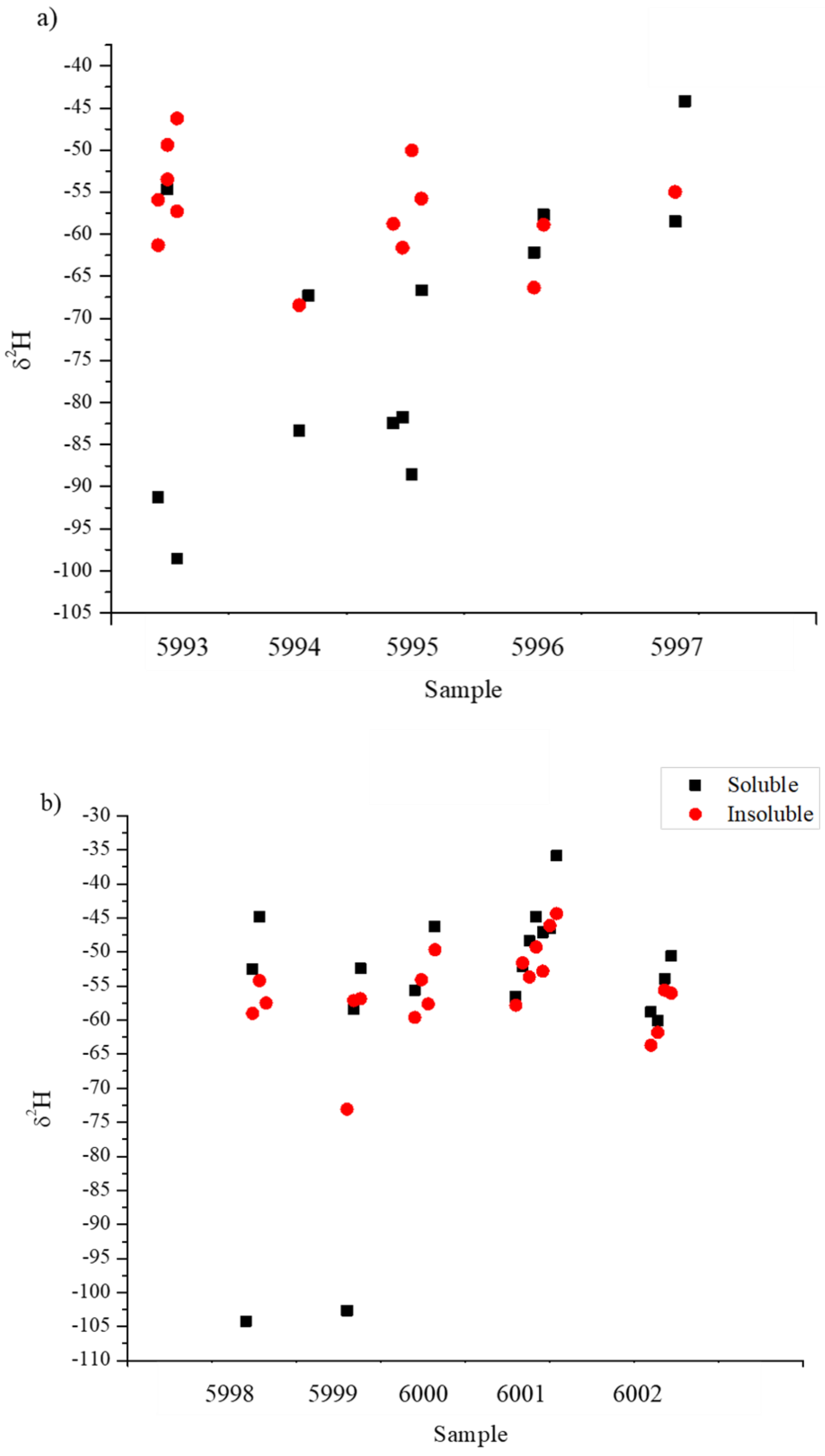

Figure S1: Hydrogen isotope ratios shown for each sample divided into subsamples from Montehano (a) and Carasa (b).

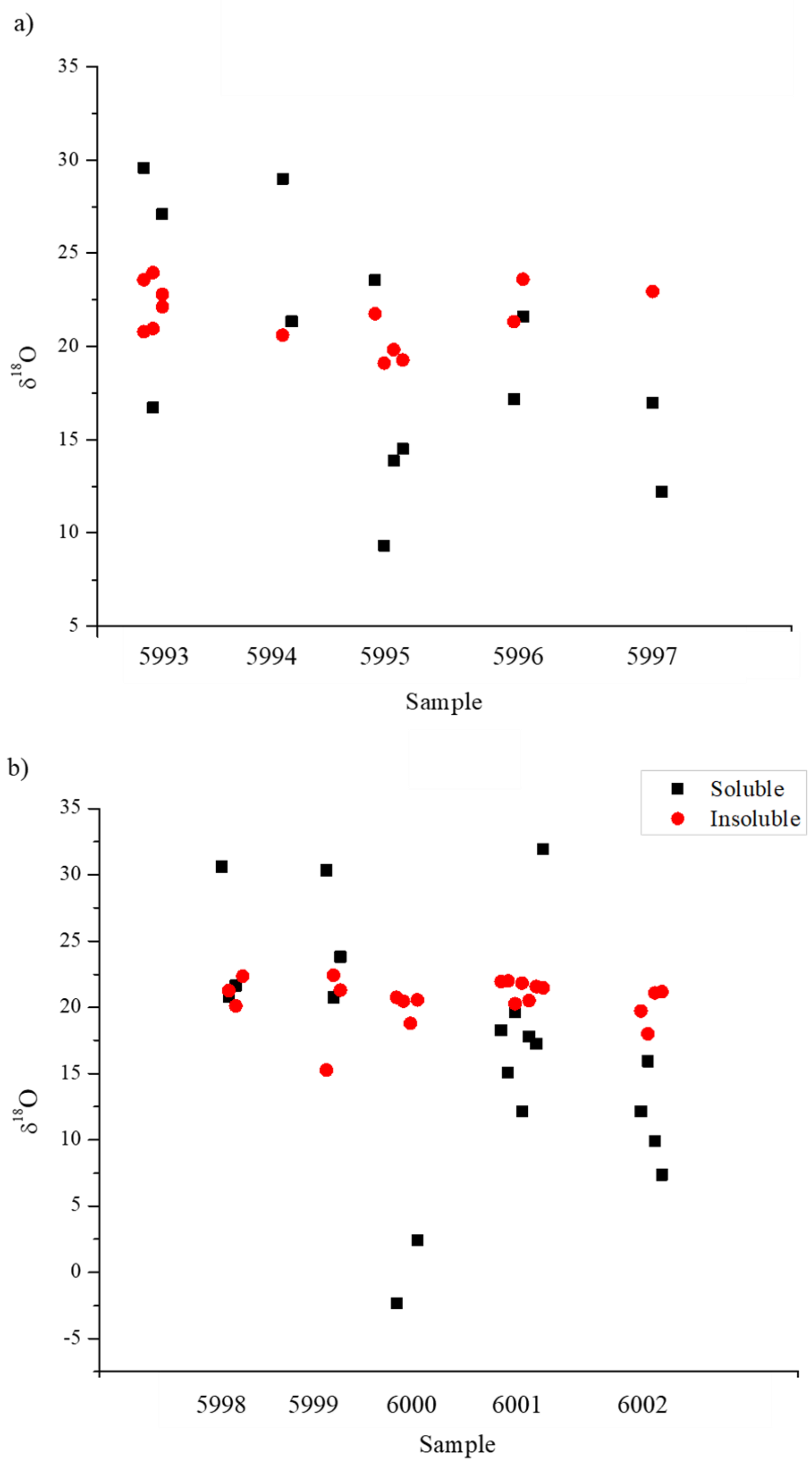

Figure S2: Oxygen isotope ratios shown for each sample divided into subsamples from Montehano (a) and Carasa (b).

Supplementary notes 2: Time series of water physical data and geochemistry

The following graphs are descriptive of the water physical data and geochemistry for each of the three sites from this study – Berria beach, lower estuary - Montehano and upper estuary – Carasa (Milano et al. 2020). They show the time series of the water salinity, temperature and oxygen isotope ratio measurements starting in December 2016 and ending in December 2017 in Berria (Fig. S3a), Montehano (Fig S3b) and Carasa (Fig S3c).

The time series following the fluctuations in time of the water temperature, salinity, and oxygen isotope values in all three sites show a strong positive correlation between salinity and oxygen isotope values (Fig. S3). Pearson's R coefficient for Montehano $R = 0.97$; for Carasa $R = 1$; and for Berria $R = 0.99$ (including the outlier on 5th of December).

The correlation between temperature and both other variables is moderate for Montehano and Carasa (Pearson's R coefficient $R \approx 0.5$ and $R \approx 0.6$ respectively), while for Berria it is weak (Pearson's R coefficient $R \approx 0.2$).

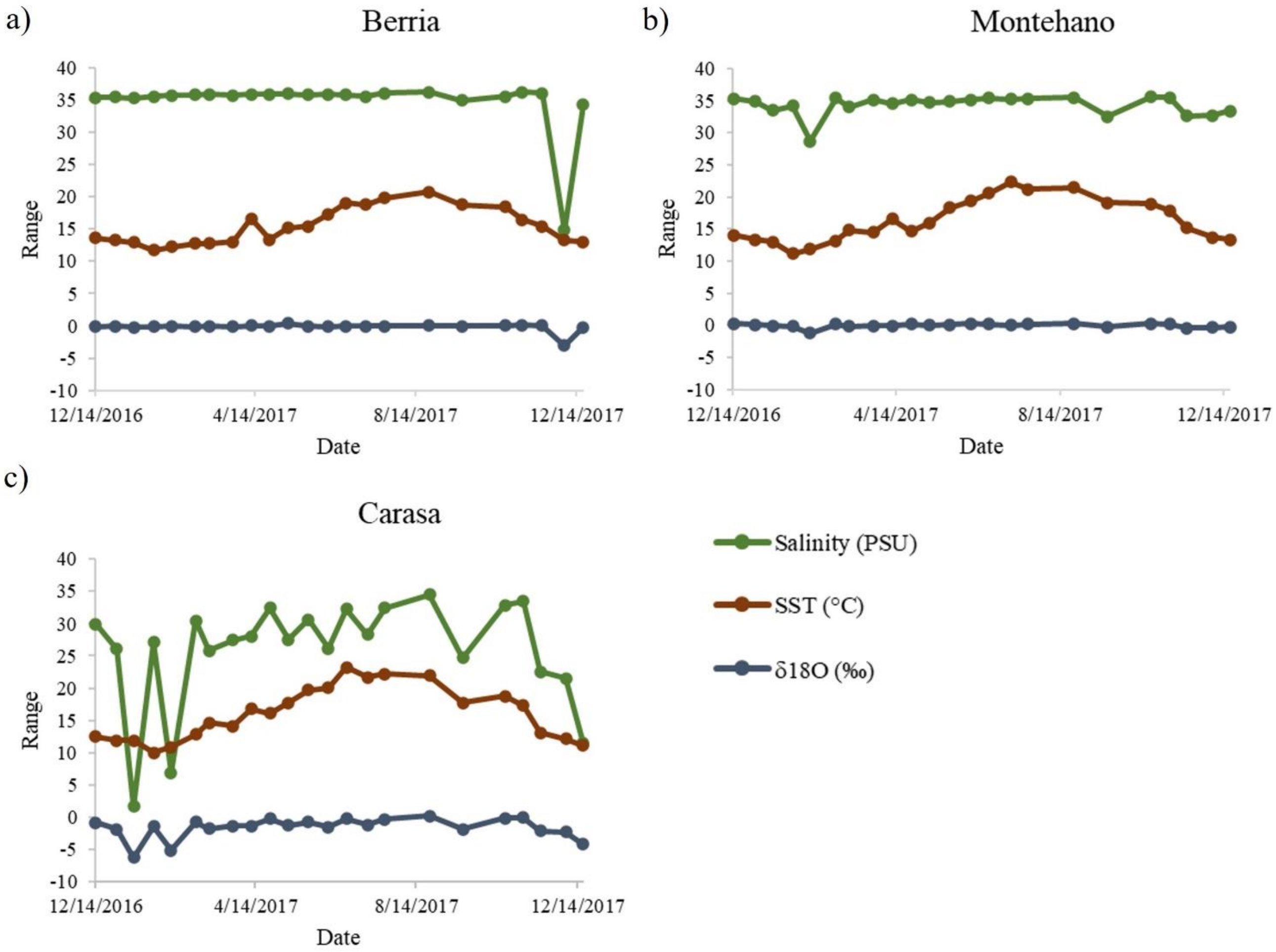

Figure S3: Time series of monthly measurements from December 2016 until December 2017 (X axis) of salinity (PSU); surface water temperature (SST in °C) and oxygen isotope values ($\delta^{18}O$ ‰) of the water in a) Berria Beach; b) Montehano; c) Carasa. Data from (Milano et al. 2020).

Supplementary notes 3: Comparison of salinity, temperature, and oxygen isotope ratios among the three sites

The salinity values among the three sites showed significant differences (Figure S4a; Kruskal-Wallis p = 7.354E-11) with a Post-hoc Mann-Whitney pairwise test revealing significant differences among all three sites.

The oxygen isotope values among the three sites showed significant differences (Figure S4b; Kruskal-Wallis p = 2.067E-07) with a Post-hoc Mann-Whitney pairwise test revealing significant differences between Carasa and both other sites.

The temperature values among the three sites showed no significant differences among the three sites (Figure S4c; Kruskal-Wallis p = 0.64).

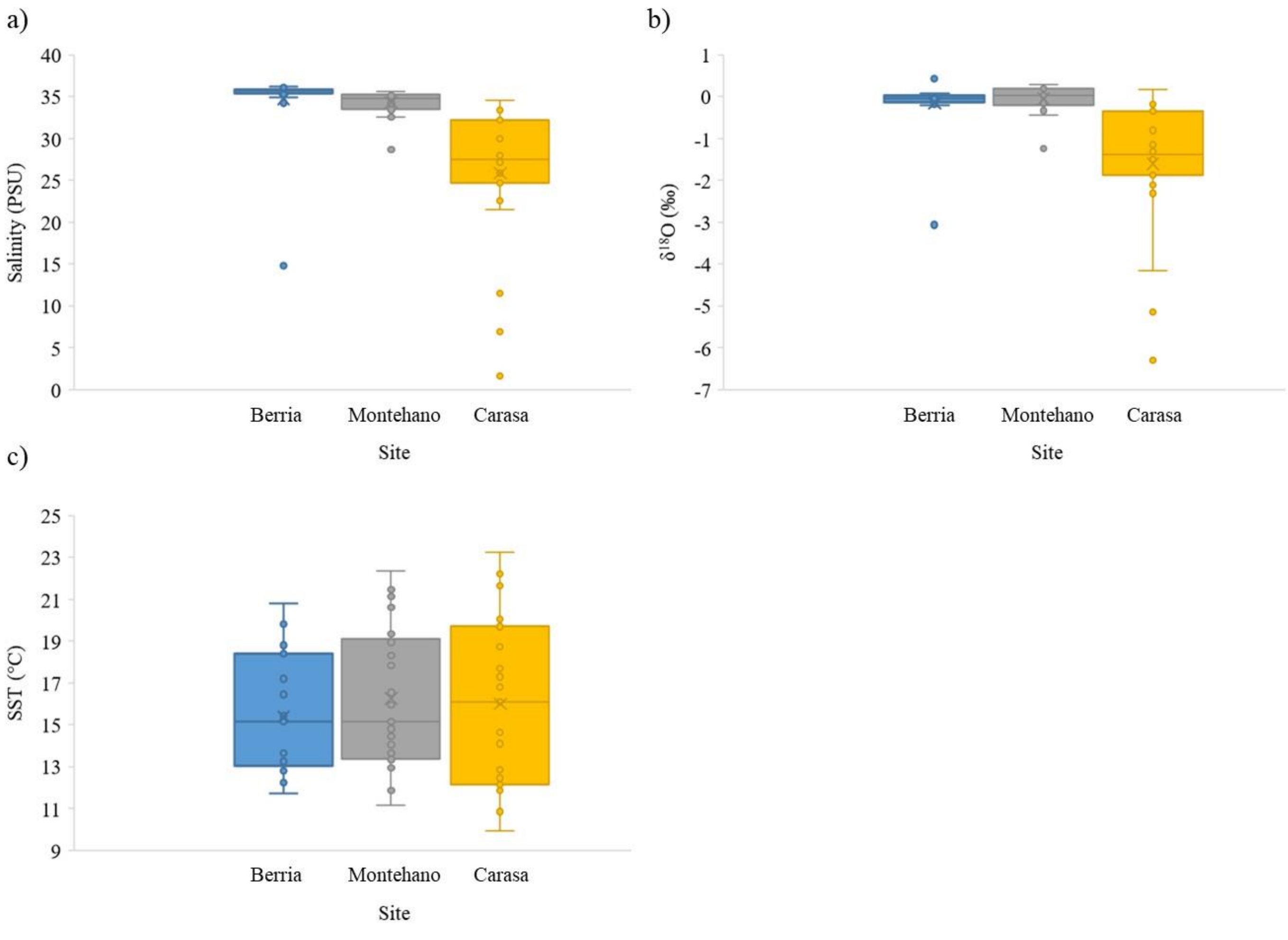

Figure S4: Boxplots showing the ranges of a) salinity (PSU); b) $\delta^{18}O$ (‰) and c) SST (°C) during the period of monthly measurements starting in December 2016 until December 2017. N=23. **** - p < 0.0001. Data from (Milano et al. 2020).

Supplementary notes 4: Generalised Linear Mixed Model analysis - GLMM

The following graphs resulted from performing GLMM analysis on the stable isotope data using the JASP software. The random effects grouping variable used was the sample number, as there were multiple datapoints for one sample for the cases where multiple subsamples were measured, and the fixed effects variable was the site. The only case where the stable isotope ratios differed significantly between the three sites was the hydrogen isotope ratio in the soluble organic matrix.

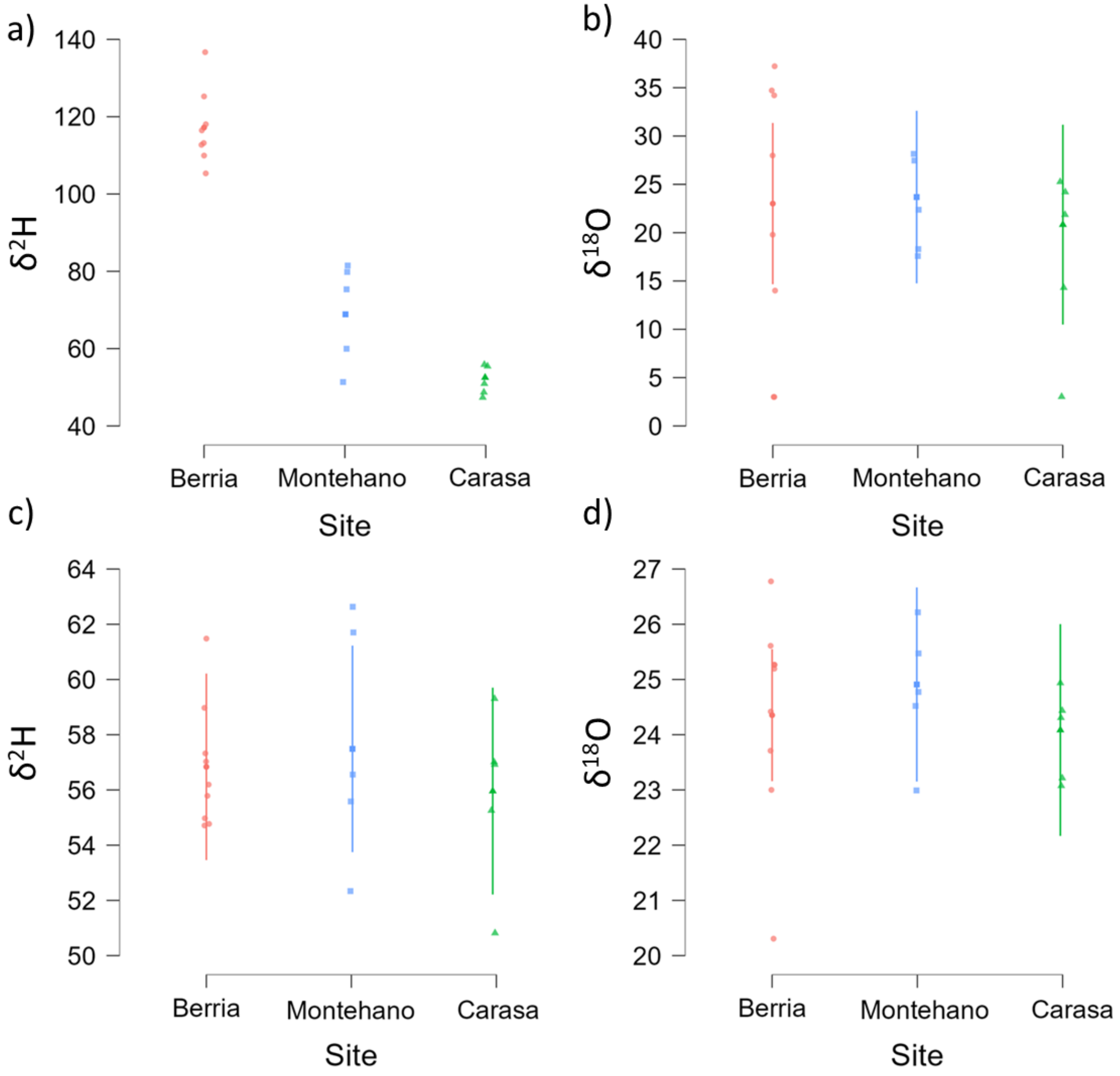

Figure S5: GLMM analyses graphs showing a) the hydrogen isotope ratios from the soluble organic matrix, $p > 0.5$; b) the oxygen isotope ratios from the soluble organic matrix, $p >0.5$; c) the hydrogen isotope values from the insoluble organic matrix, $p < 0.001$; and d) the oxygen isotope ratios from the insoluble organic matrix, $p > 0.5$.

Supplementary References:

Milano S, Schöne BR, Gutiérrez-Zugasti I. 2020. Oxygen and carbon stable isotopes of mytilus galloprovincialis lamarck, 1819 shells as environmental and provenance proxies. The Holocene. 30(1):65-76.

JASP Team (2023). JASP (Version 0.17.3) [Computer software].
