# Supplementary material for: Author Correction: Stable isotopes in the shell organic matrix for (paleo)environmental reconstructions
Source: Commun Chem. 2025 Oct 28;8:316. doi: 10.1038/s42004-025-01745-2 (PMC12569266; doi:10.1038/s42004-025-01745-2)
Supplement: Supplementary file 1 — Original, uncorrected Fig. 4 [file 42004_2025_1745_MOESM1_ESM.pdf]

Original, uncorrected Fig. 4

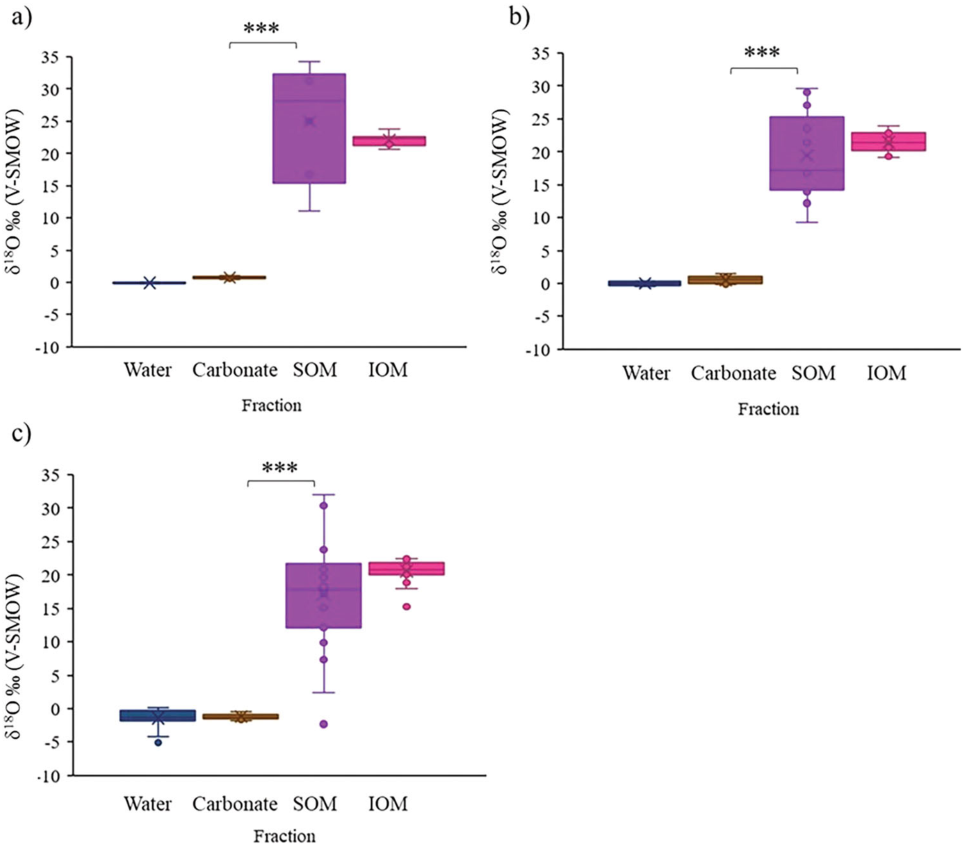

Fig. 4 Comparison of the oxygen isotope values of the water, carbonate, and the soluble and insoluble organic matrix fractions. Boxplots showing the $\delta^{18}O$ of the water, the shell carbonate as well as the Soluble Organic Matrix (SOM) and Insoluble Organic Matrix (IOM) phases for all samples from **a** Berria. $N = 6$; **b** Montehano. $N = 13$; **c** Carasa. $N = 19$. *** = $p < 0.001$. The x represents the mean, the median value is shown as the line and outliers are given as the points outside of the whisker ranges. Water and carbonate data from Milano et al. (ref. [48]; Table 1; Page 69).
